# Emergency department reorganisation to cope with COVID-19 outbreak in Milan university hospital: a time-sensitive challenge

**DOI:** 10.1186/s12873-021-00464-w

**Published:** 2021-06-28

**Authors:** A. Jachetti, G. Colombo, B. Brignolo-Ottolini, J. Franchi, M. Solbiati, M. Pecorino Meli, P. Bosco, G. Costantino

**Affiliations:** 1 UOC Pronto Soccorso e Medicina d’Urgenza, Fondazione IRCCS Ca’ Granda Ospedale Maggiore Policlinico, Milan, Italy; 2 Dipartimento di Scienze Cliniche e di Comunità, Università degli Studi di Milano, Milan, Italy

**Keywords:** Emergency department, COVID-19, Management

## Abstract

**Background:**

In March 2020 we faced a huge spread of the epidemic of SARS-CoV2 in northern Italy; the Emergency Departments (ED) and the Emergency Medical Services (EMS) were overwhelmed by patients requiring care. The hospitals were forced to reorganize their services, and the ED was the focal point of this challenge. As Emergency Department in a metropolitan area of the region most affected, we saw an increasing number of patients with COVID-19, and we made some structural and staff implementations according to the evolution of the epidemic.

**Methods:**

We analysed in a narrative way the weaknesses and the point of strength of our response to COVID-19 first outbreak, focusing point by point on main challenges and minor details involved in our ED response to the pandemics.

**Results:**

The main stems for our response to the pandemic were: use of clear and shared contingency plans, as long as preparedness to implement them; stockage of as much as useful material can be stocked; training of the personnel to be prepared for a fast response, trying to maintain divided pathway for COVID-19 and non-COVID-19 patients, well-done isolation is a key factor; preparedness to de-escalate as soon as needed.

**Conclusions:**

We evaluated our experience and analysed the weakness and strength of our first response to share it with the rest of the scientific community and colleagues worldwide, hoping to facilitate others who will face the same challenge or similar challenges in the future. Shared experience is the best way to learn and to avoid making the same mistakes.

## Background

In November 2019, an outbreak of a Novel Coronavirus (alternately COVID-19, COVID or SARS-CoV-2) was discovered in the Hubei Region in China. Despite the preventative measures taken by Chinese Health and Political Authorities, the virus spread across China and neighbouring countries. It arrived undetected in Europe in January 2020. The World Health Organization (WHO) issued an international alert on January 5, 2020. (https://www.who.int/csr/don/05-january-2020-pneumonia-of-unkown-cause-china/en/) During the following month, Northern Italy faced a massive spread of the epidemic. Emergency Departments (ED) and Emergency Medical Services (EMS) were overwhelmed by patients requiring urgent care [1, 2]. Hospitals were forced to reorganize their services, such as Intensive Care Units [3, 4] but the ED was always the main stem of this challenge. Others Authors share their experiences in different settings [5, 6], this paper reports the experience of *Fondazione IRCCS Ca′ Granda Ospedale Maggiore Policlinico* hospital in reorganizing a major metropolitan emergency department in response to new, challenging needs related to COVID-19.

This paper also reports our hospital’s response to a challenging situation: doing our best to deal with a considerable number of severe respiratory patients while trying to maintain a safe workplace for all healthcare workers and non-COVID-19 patients.

### Facility

The *Fondazione IRCCS Ca′ Granda Ospedale Maggiore Policlinico* is a University Hospital in Milan. With more than 800 beds divided across various buildings, it is one of Milan’s largest and busiest public general hospitals. Its ED sees approximately 75,000 patients per year. The Emergency Department is a referral center for trauma, neurological, neurosurgical and cardiovascular disease. During a typical day’s shift, five doctors, eleven nurses and three assistants are on duty. As a University hospital, residents in Internal Medicine and Emergency Medicine are present at all times. Before the COVID-19 outbreak, the ED was organized with a separate triage for walk-in patients and EMS, then subdivided based on the required care intensity. Subdivisions included:

- A four-patient shock room adjacent to the triage room, stocked with ventilators and equipment to treat severe patients.
- An initial evaluation unit with three examination rooms and 14 beds for patients with low- or medium-intensity of required care.
- Four examination rooms with dedicated waiting area for walk-in patients with the lower intensity of required care.
- Special fast-tracks for minor surgical needs and otorhinolaryngoiatric or ophthalmological emergencies during the daytime.
- Pediatrics, gynecological and dermatological emergencies had their dedicated emergency rooms in their designated ward.
- Psychiatric emergencies had dedicated rooms and staff 24-h per day.
- A large short-stay observation room (OBI) with 14 beds and additional staff dedicated to intensive- and fragile- care patients; the OBI also served as a waiting room for patients waiting for hospitalization, with four additional beds in smaller rooms.
- Two isolation rooms were also available for patients with suspected infectious diseases or who need special protection.
- Two other rooms were ordinarily available for special services such as domestic violence interviews or private communication with the patients’ families.
- On the second floor, aside from the surgery department, a 10-bed high-turnover ward was implemented in December of 2019 for patients who require low-severity hospitalization or observation. The whole department had a dedicated radiology service with echography, CT-scan, and X-rays operating 24 h a day. Interventional radiology procedures were available on call.

## Initial response to COVID-19 outbreak

On January 23, 2020, before the Regional Health Authorities indication of an outbreak in Lombardy, *Policlinico* planned a dedicated pathway for patients with suspected COVID-19. Those measures were implemented on January 25, providing a means to isolate patients with symptoms associated with a SARS-CoV-2 infection (fever with tiredness, dry cough, aches and pains, nasal congestion or sore throat) with an epidemiological link to Wuhan or the Hubei region. Due to the small number of suspected patients, the isolation rooms were sufficient to deal with the challenge. At that point, the waiting time for the results of a SARS-CoV-2 testing swab was between 16 to 24 h. Ideally, patients in isolation should have worn surgical masks, yet Personal Protective Equipment (PPE) was available only for healthcare professionals in direct contact with patients.

In early February, following the spread of the virus from the Hubei Region to most of China, the epidemiological criteria to isolate patients changed. After the first locally transmitted case of COVID-19 in Codogno, a small town close to Milan, on February 21, it was again changed from China to all the ten municipalities related to the new outbreak in Italy. The numbers of suspected cases increased dramatically, while confirmed cases still were not found in our ED at that time.

Due to the long waiting time for the results of SARS-CoV-2 swab tests, our laboratory on February 26 was prepared to internally process the swabs, reducing the evaluation time to four to six hours. By this time, the influx of patients with suspected COVID-19 was too high to be handled by just four dedicated rooms. Every suspected cases needed isolation to protect other patients and other suspected cases from the infection. The ED, organized in large rooms where plastic curtains could grant a bit of privacy to patients, even if it was not nearly enough to protect patients from the virus. So, the surgery department was rapidly moved to another building and the ward adapted to receive suspected COVID-19 patients, with previously double rooms transformed in single isolation room and full cleaning and proper disinfection after every patient.

**Fig. 1:**
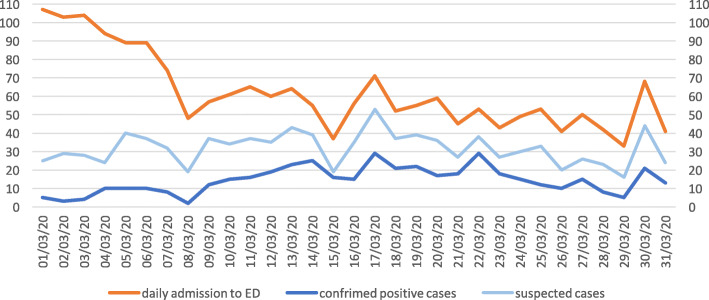
Daily admission in *Policlinico* Emergency department on March 2020

**Fig. 2:**
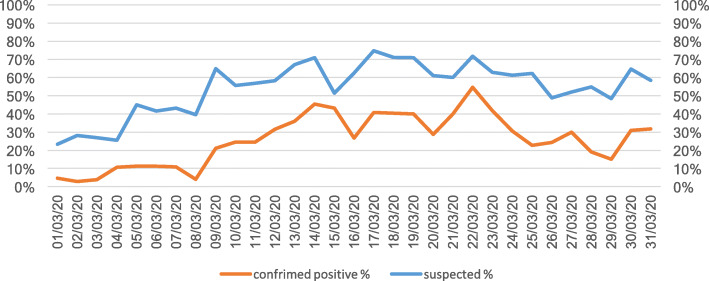
Suspected and confrimed COVID-19 cases (%) in *Policlinico* Emergency department on March 2020

As long as the epidemics spread through the people in Lombardy region, our triage criteria for suspected COVID-19 infection changed. By the end of February, all suspected COVID-19 cases identified in triage, based to the sole presence of typical symptoms while the epidemiological criteria were considered obsolete and definitely removed, were received directly at the ward. We also transformed the EMS entrance to the ED into a dedicated entrance for suspected COVID-19 cases. After a quick triage with all staff wearing full PPE, the suspected cases were sent to a nearby dedicated lift to the second floor, where full PPE operators, alerted by colleagues in triage, were prepared for the arrival of suspected patients. All patients entering the second floor ward were assigned to a single room with a total capacity of 24 patients at the floor. Patients that tested positive were put together in double rooms while awaiting transfer to a dedicated COVID-19 department. This solution was immediately effective on patients overcrowding in the ED. This approach permitted to keep the rest of the ED to remain clean and operative for non-COVID patients, who still entered the ED through the original walk-in entrance. Noticeably, the volume of non-COVID patients diminished each day, see Figs. 1 and 2.

Unfortunately, severe respiratory patients could not be admitted and treated directly at the ward on the second floor, considering that those rooms were not yet ready for high-intensive care. So, at the ground floor inside the ED, the isolation rooms and the two small rooms nearby to the observation area were transformed into a new intensive area were severe respiratory patients with high suspicion of COVID-19 infection could be treated. The shock room remained clean for trauma and other severe patients not suspected to have COVID-19 related conditions.

Essentially, in March, *Policlinico* split its facility into two different emergency departments:

- The COVID-19 pathway was given its own entrance, triage, and high-intensity area with six positions in the isolation area and 24 admission and observation isolation rooms;
- The non-COVID pathway was given its own entrance, triage, shock room, the initial evaluation room for admission and the short-stay observation unit main room for observation and recovery.

During this time, to accommodate growing COVID-19 activity, dedicated departments were opened throughout the hospital. Surgery activity was reduced to emergencies, as were ambulatory visits and visits to clinics. Still, and despite the increasing capacity for COVID-19, the severity and the sheer number of patients were high. Not all who required treatment in the Intensive Care Unit (ICU) could be accommodated. Implemented strategies and protocols came to a break-even point and then beyond, where available resources and patients’ needs could not meet.

## Late response

**Fig. 3:**
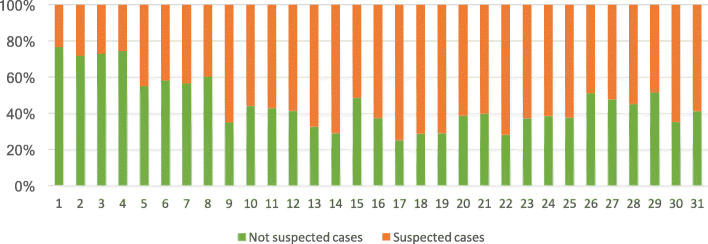
Supected and non-suspected COVID-19 cases (%) in *Policlinico* Emergency department on March 2021

By mid-March 2020, our surge capacity was at its limit. All beds were full without any possibility of transferring patients to other facilities due to the full capacity of all the hospitals in Lombardy region. During the second and third week of March, the outbreak hit its peak. National lockdown measures taken by the Government on March 10 were not yet effective and the number of patients with suspected COVID-19 was impossible to be managed at the second floor ward. During that time, the influx of COVID-19 patients forced us to use the short-stay observation room to increase our intensive respiratory admission room capacity. Non-COVID patients flow was at its nadir (see Fig. 3). The hospital capacity for inpatient COVID-19 patients reach the limit. Waiting time for hospital admission was very long and the first nurses and doctors affected by COVID-19 reduced our capacity to open new beds. New semi-intensive care units were urgently needed to admit COVID-19 respiratory failure patients; the entire ED was lined with patients on stretchers which could not be admitted nor discharged.

### New setting

Considering that scenario, as anticipate in our contingency plan, we decided on March 11 to make a switch. We moved the non-COVID patients in isolation area, which was attended by general and emergency surgeons, and the rest of the ED became the COVID-19 emergency room, attended by ED personnel. Patients arriving at the ED with non-COVID symptoms were sent to an isolation area after entering through an available backdoor. We went back to the normal pathway for COVID-19 patients, who were divided according to their severity. Staff wore full PPE for their entire shift. Essentially, we returned to a walk-in clinic for COVID-19, with basic nurse protocol to perform a blood test and ask a chest X-Rays. Orthopaedic surgeons were in charge of those patients, they were specifically trained to perform ABG analysis, walking-test and lung ultrasound, stratifying their risk. The evaluation room was returned to patients on stretchers with suspected COVID-19, while the full short-stay observation room became the intensive respiratory unit for COVID-19 patients. The ward on the second floor was transformed from an admission area to a hospitalization ward for high-intensive patients, a sort of semi-intensive care unit run by ED personnel. Doctors and nurses returned to the resources they were used to, but the influx of COVID-19 patients required help from colleagues brought in from the rest of the hospital. This change gave relief to all the patients waiting to be hospitalized, providing immediate accommodations, especially for the most severe cases.

### Invent new spaces

Unfortunately, those changes were not enough. A week after, still under pressure from the influx of COVID-19 patients, we moved to another phase of our contingency plan to open the main waiting room outside the triage. At the beginning of the outbreak, this room was furnished with chairs, benches and vending machines and usually filled with families and patients waiting to be called. Since the beginning of the emergency, the contingency plan called for it to be transformed into something more useful. The hospital’s clinical engineers were given the mandate to prepare 14 oxygen nozzles linked with the hospital oxygen distribution grid. This way, when the Emergency Department once again became too crowded, we created a new room for severe respiratory patients. This bought time for the rest of the hospital and for the Regional Health Authorities to develop new places to transfer and hospitalize low-intensity COVID-19 patients.

### The flat curve

In the second half of March 2020, we achieved a slow but progressive reduction of the influx of COVID-19 patients. The surge passed and we were better equipped to manage patients in terms of flows and bed management. In April 2020, even the ICUs started transferring their patients. COVID-19 departments were still active but able to manage the new patients coming from the ED. In Table 1 the evolution of our response to many issues in different pandemic phases.

**Table 1:** Evolution of ED response through epidemic phases

Issues	Initial response	Late adaptation	Definitive set-up
*Triage*	Single	Separated	Separated
*Triage criteria*	Isolation for people with symptoms arriving from Wuhan and Hubei region	Isolation for people with symptoms arriving from the affected area in Italy	Isolation for people with suspected symptoms, no geographical limitations
*Isolation rooms*	4 to 6 in ED for suspected cases	24 on the floor for suspected cases	Four at ED for protection or immunodeficiency
*Walk-in COVID patients*	Direct to isolation rooms	Direct to isolation rooms	A dedicated area in ED with four rooms
*Severe COVID patients*	Direct to isolation rooms	A dedicated area in ED with four rooms	A dedicated area in ED
*Non-COVID patients*	Full ED	Waiting area repurposed with 14 beds	Previous isolation area with 8 bed places
*PPE*	Surgical mask, but full PPE only in isolation rooms	Full PPE everywhere	Full PPE everywhere
*Swab waiting time*	16 to 24 h	10 to 12 h	4 to 8 h
*ED personnel*	Normal set-up	Normal set-up in ED + Surgery Nurses and 2 ED doctors at floor	Normal staff in COVID ED + 2 nurses + other specialists (1 or 2 per shift) in walk-in area + Surgery Nurses and 2 ED doctors + 1 other specialist at floor

## Other aspects

### Dealing with families

Even during normal activities, the relationship between physicians and patients’ families is crucial in the ED. By nature of the ED, high patient volume, limited time, and high pressure are not ideal for dealing with families. From a family’s perspective, spending time with their hospitalized relative and obtaining news and information directly from doctors is expected. Before this epidemic, a family member was always permitted to stay inside the ED with fragile patients, while a large waiting room was dedicated for the extended families or caregivers. In this outbreak, the risk of contagion among family members and patients forced us to keep relatives away from the ED and the ward. PPE, isolation and extremely limited time changed the dynamics of family communication. Dying patients were not able to see their relatives. In turn, the patients’ stress was higher because of forced isolation and because they did not have the face-to-face support of their relatives. We set up a strategy to cope with families based on regular phone calls. We first enlisted medical students who were already familiar with the team and our hospital software. Then a dedicated phone number for relatives was set up, with medical students covering the phones in two shifts from 8 AM to 8 PM providing general information. The students also participated in our meetings during changing shifts, and they followed the state of the patients on clinical software. However, main clinical information was provided only by the physician responsible for the patient. Electronic tablets were donated to our department to organize regular video-calls among family members. These strategies were well-received and appreciated by families and patients.

### Training and formation

The ED staff alone were not prepared to deal with the massive influx of patients during the emergency. As in a Mass Casualty Event (MCE), where human and technical resources can be moved rapidly, we could find help inside our hospital to handle this crisis. Unlike a usual MCE, however, this emergency lasted for many weeks, not for hours, and the personnel was at risk of contracting COVID-19. The ED staff was not able to handle this crisis alone, and at the same time, it could not allow leaving other specialities colleagues unprepared to face the challenges posed by the outbreak.

We trained an emergency staff on practical skills and theoretical information. We started with PPE use and disposal, passing to arterial-blood gases (ABG) interpretation, basic lung ultrasonography skills arriving at basic ventilation skills in COVID-19 patients. A one-hour lesson on basics about COVID-19, always with social distancing measures, followed by practical workshops in small groups, demonstrated to be effective. We also prepared flow charts and protocols to speed up and simplify evaluations, risk-assessments, and appropriate treatments that satisfied our team and the host colleagues, who acquired skills that can be useful in the future. Resident doctors were a considerable part of the response. They also were trained, and they were able to cope with many patients and strengthen our capacity to respond.

## De-escalation

After the outbreak’s surge, the same flexibility and quick response were applied to the de-escalation. De-escalation should be anticipated for resources, personnel and facility, and it should be planned as soon as possible and implemented at the right time. There are no clear benchmarks to follow. Like preparing for escalation, de-escalation needs to be based on how any particular ED is organized and how resources are allocated. A de-escalation plan should be written and shared with all the actors involved, just as a contingency plan for an outbreak.

In our scenario, during de-escalation, we attended to a reduced flow of COVID-19 patients daily. We decided to switch the isolation area to COVID-19 patients and bring non-COVID patients to the waiting room, ready to host up to 14 stretchers. Of course, sanitizing all the equipment and the room was a prerequisite to the switch. The walk-in area was also transformed into a non-COVID area, using the four rooms as an observation room. De-escalation also meant that colleagues sent to help at the ED (while their units were closed) could be reassigned to their original services. Shifts were modified week by week to better cope with the changing situation.

Experience in China shows that new outbreaks can be linked to returning residents or asymptomatic spreaders [7], so cautiousness is recommended before reopening non-COVID services and policies. Strict isolation and preventive measure should be maintained in subsequent weeks, following WHO indications on the COVID-19 trends worldwide (https://www.who.int/emergencies/diseases/novel-coronavirus-2019/technical-guidance).

De-escalation is not only related to practical issues but also on a psychological and emotional approach to the personnel involved in the response. After weeks with a high-stress level and focusing only on a cataclysmic medical situation, ED physicians could benefit from seeing and treating patients with different conditions. Yet caution should be urged, as the trauma of dealing with a pandemic could, psychologically, cause physicians to underestimate other pathologies that less severe than COVID-19.

## Challenges

This emergency has been a substantial challenge for our country, our health service and our hospital. We made mistakes, from which we learned, but we also faced something modern society has never confronted with.

One lesson learned is that our response’s weakness could be assigned first to our lack of preparedness. After the major crisis in China, we did not immediately prepare a proper contingency plan to deal with such a monumental challenge, whether national, regional, in the hospital, or a specific hospital department. We prepared in the past many contingency plans for mass casualty events. We trained and studied, but no one ever planned for anything this hard and this long. Facilities, sadly, were not studied and designed to respond to this kind of emergency.

Other weaknesses are related to the use of PPE, too often considered a tedious preventive measure. Yet, this time, its proper use and availability saved hundreds of healthcare workers. Our lack of PPE at the beginning of the outbreak created a situation where there was not enough for all, and some of our personnel were, as a result, affected by the virus. Even if a doctor or nurse were not in danger of dying from COVID-19, they were not available to help with the cause for weeks, and, unfortunately, the first to fall are the more experienced and skilled practitioners who cannot lend their services during the rest of the emergency.

Another notable weakness was the lack of measures to prevent contagion in non-COVID departments. The use of PPE and isolation was limited. When personnel and patients with no signs of SARS-CoV-2 infection introduced the virus, spreading contagion and forcing the administration to close and sanitize huge wards. This lack of personnel and consequential extra shifts, limited also research and scientific work during the pandemic: a missed opportunity for a University Hospital.

We also found that even with multiple nasopharyngeal swab test negative for COVID-19, strongly suspected cases should be kept in isolation and a bronchoalveolar lavage specimen for COVID-19 detection should be obtained as soon as possible. Often, a first negative swab did not exclude COVID-19, which was later confirmed by further analysis By the way, those patients could not be recovered with positive cases neither with negative, so they needed a special hospitalization ward with isolation rooms.

Those and other challenges we faced are listed in Table 2 below.

**Table 2:** main challenges in the outbreak and response

Challenges	Response
*Lack of PPE, misuse and theft*	Increased order, closed stock and counted distribution to personnel according to their shifts.
*Patients with clinical suspect of COVID19 with a negative swab test*	CT scan and double swab test in high clinical suspicion, if still negative swab on bronchoaspirate or bronchoalveolar lavage.
*Lack of isolation rooms*	Early adaptation of a department floor (24 rooms) in ED isolation area with the possibility to transform into double rooms.
*Food delivery*	Pre-ordered closed meals to distribute to each single patient
*Laundry and scrubs for staff*	Single-use paper scrubs and new unnamed scrubs with daily change in dedicated stock, special biocontainment bags for used scrubs with dedicated laundry system.
*Cleaning and sanitizing*	Implemented a 24 h/24 cleaning staff inside COVID-19 area.
*Lack of oxygen nozzles*	Oxygen nozzle splitters can double each nozzle, flow-meter up to 30 Lit/min.
*Lack of CPAP devices*	Increased order of masks and Venturi device, use of resin 3Dprinted Venturi device that can be used with standard oxygen nozzles.
*Understaffing*	Training to other specialists and staff to be assigned to walk-in patients or low-intensity departments with clear and shared protocols to assess COVID-19 patients and senior ED physician supervision.
*Information and contact with families*	Implementation of a daily routine call system with VoIP or other communication devices. Systematic routine phone calls daily by the physician in charge. Dedicated phone number for families overseen by Medical Students to provide information.

## Take home messages and suggestions

The most valuable takeaway is preparedness. Wherever the virus has not yet arrived, start training personnel and drawing contingency plans. Collect materials and equipment before it is too late. Value and enhance teamwork: it is effective in caring for patients and coping psychologically with such enormous challenges for healthcare workers. We learned to be creative, such as printing in 3D resin made Venturi-meters to bring CPAPs in every ward and reconfiguring the original emergency department to make isolation rooms. We learned how important the role of the healthcare workers, suppliers, cleaners and clinical engineers is to support our daily work. These key people have to be protected and supported, or there will be no success in overcoming the pandemic’s challenges. We learned that data and the capacity to interpret them are fundamental in anticipating the response and understanding where to allocate resources. Never stop collecting data and never stop reading and analysing them properly. The final lesson learned how undervalued communication could sometimes be. Communication inside the team, among physicians, services, decision-makers, health authorities and families, all communications is fundamental to reduce the risk of errors and improve the climate among all actors.

In summary, some suggestions to colleagues facing this challenge are:

1. Make contingency plans to be implemented according to number of daily accesses and severity of patients, share it with medical direction and other departments
2. Stock as much ventilation material as is possible and train all personnel to use it
3. Stock as much as PPE as you can and train all your personnel to use it
4. Prepare basic flow-charts and train your personnel, even the one normally not used to work in ED
5. Prepare to be flexible in your response and ask for flexibility from colleagues, other departments and facility managers
6. Try to divide pathways as much as you can; isolation is the key
7. Treat all the suspect COVID-19 patients as if they should test positive; do not always rely on swabs
8. Be prepared to de-escalate when conditions change

## Conclusions

As we see by the recent second and third wave, COVID-19 is far from concluding worldwide. The logistical, emotional and psychological burden of deaths, dedicated resources, and over-stressed health facilities, will last for a long time. We evaluated our experience and analysed the weakness and strength of our first response to share it with the rest of the scientific community and colleagues worldwide, hoping to facilitate others who will face the same challenge or similar challenges in future. Shared experience is the best way to learn and to avoid making the same mistakes.

## Data Availability

Data is property of Fondazione IRCCS Cà Granda Ospedale Maggiore Policlinico Milano. The datasets used and analysed during the current study are available from the corresponding author on reasonable request and upon approval of the health board of Fondazione IRCCS Cà Granda Ospedale Maggiore Policlinico Milano.

## Abbreviations

ABG: Arterial Blood Gas
CPAP: Continuous Positive Airway Pressure
CT: Computer Tomography
ED: Emergency Department
EMS: Emergency Medical Services
ICU: Intensive Care Units
IRCCS: Istituto di Ricerca e Cura a Carattere Scientifico (Public—funded research hospital)
MCI: Mass Casualty Incident
PPE: Personal Protective Equipment
WHO: World Health Organisation
